# Validations of verbal autopsies – comment on ‘automated versus physician assignment of cause of death for verbal autopsies: randomized trial of 9374 deaths in 117 villages in India’

**DOI:** 10.1186/s12916-019-1371-0

**Published:** 2019-07-16

**Authors:** Michel Garenne

**Affiliations:** 1 Institut Pasteur, Epidémiologie des Maladies Emergentes, Paris, France; 2 MRC/Wits Rural Public Health and Health Transitions Research Unit, School of Public Health, Faculty of Health Sciences, University of the Witwatersrand, Johannesburg, South Africa; 3 Institut de Recherche pour le Développement (IRD), UMI Résiliences, Bondy, France; 4 FERDI, Université d’Auvergne, Clermont-Ferrand, France

**Keywords:** Verbal autopsy, Validation, Automated diagnosis

## Background

Verbal autopsy (VA) is a method for assessing causes of death by interviewing relatives of a diseased person and gathering as much information as possible on the diseases, signs, symptoms, treatments, and circumstances of the death. The gathering of such information can be made by informal interviews or through the use of a questionnaire, and is then treated either by knowledgeable persons (e.g., trained physicians) or by a computer in order to obtain the probable causes of death (underlying, immediate, associated). VA using a standardized questionnaire was first used on a small scale in the 1980s and became popular in the 1990s [1, 2]. From the beginning, the issue of validation was crucial. The reliability of the final assessment of the individual cause of death or of the distribution of deaths by cause are two different, though closely related issues, which require separate interpretations and have different implications.

The first issue – the reliability of individual cause of death – is assessed by comparison with a ‘gold standard’. Several ‘gold standards’ have been used over the years, in particular ‘clinical diagnosis’ made by physicians in hospitals and based on clinical and biological examinations as well as ‘formal autopsies’ based on postmortem histopathological examination. The determination of the best method can be discussed at length. From a purely theoretical standpoint, comparison with a formal autopsy is the most robust but has serious limitations. Firstly, formal autopsies are rarely conducted except in the case of violent or suspicious deaths, which is not a representative sample of all causes of death in the population. Secondly, the precise assessment of all pathological processes leading to death is sometimes far from the ‘underlying cause’, the concept used in public health. From a practical standpoint, assessing the underlying cause, as done in developed countries, is based on a mixture of clinical and biological examination, knowing that those are not identical to formal autopsies [3, 4]. Therefore, VA diagnoses are expected to fit as closely as possible these ‘underlying’ causes of death. If VA diagnoses were 100% sensitive and specific compared with clinical diagnoses, then the distribution of causes of death obtained from VAs would be identical to that obtained from clinical diagnosis. Even if sensitivity and specificity were not 100%, the distribution of causes obtained by VAs would be close to that obtained by clinical diagnosis, assuming that sensitivity and specificity are high enough. Furthermore, even if not perfect, when VAs are consistent over the years, changes in the cause of death structure revealed by the VAs are likely to fit real changes in the population, which is the most important point for public health purposes (measuring progress or identifying emerging issues). The use of VAs raises numerous technical problems. For example, the list of causes targeted by VAs, which represent the leading causes of deaths important for public health purposes, is context specific and varies across countries; some of these causes may not be assessable by VAs; the quality of VAs may vary according to the questionnaire used, to the physicians reading them, or to the algorithm used for their interpretation, etc. Therefore, the value of VAs may vary considerably between studies and the results need to be interpreted with caution.

The paper presented by Jha et al. [5] has an aim that differs from classic validation, namely to compare the distribution of causes of death obtained by human (physician) assignment with that obtained by computer (automated) diagnosis (six algorithms were tried), called ‘population-level concordance’. The authors go even further – they do not compare causes of death based on the same individual cases, but they compare two different datasets, randomly assigned, assumed to provide the same distribution of causes. This approach therefore multiplies the potential biases, including differences between the two samples, differences between the diagnosis method, and differences between the algorithms, therefore complicating interpretation.

However, their study still has some value given that it is based on large numbers, all ages, and a variety of causes. In particular, it shows that, in 83% of cases, the diagnosis for adults made by two independent physicians was identical, which is reassuring. Even if both could be wrong in some cases, this at least ensures consistency. The fact that automated algorithms were shown to be often inconsistent implies that they should be improved. In fact, they are based on the same type of evidence and should lead to the same, or at least a compatible, diagnosis. There is much work to be done to improve questionnaires, coding, and automated diagnoses in order to enable the use of VAs on a large scale in countries without proper cause of death registration. Of course, ultimately, one would like to have appropriate cause of death statistics worldwide.

## Data Availability

Not applicable.

## References

[1] Garenne M, Fontaine O (2006). Assessing probable causes of deaths using a standardized questionnaire. A study in rural Senegal. Bull World Health Organ.

[2] Kahn K, Tollman SM, Garenne M, Gear JSS (2000). Validation and application of verbal autopsies in a rural area of South Africa. Tropical Med Int Health.

[3] Goldman L (1984). Diagnostic advances versus the value of the autopsy. 1912-1980. Arch Pathol Lab Med.

[4] Roulson J, Benbow EW, Hasleton PS (2005). Discrepancies between clinical and autopsy diagnosis and the value of post-mortem histology; a meta-analysis and review. Histopathology..

[5] Jha P, et al. Automated versus physician assignment of cause of death for verbal autopsies: randomized trial of 9374 deaths in 117 villages in India. BMC Med. 2019. 10.1186/s12916-019-1353-2. 10.1186/s12916-019-1353-2 PMC6595581 31242925

